# Genes associated with genetic and rare lung diseases and the risk of lung cancer

**DOI:** 10.1186/s12885-026-15934-2

**Published:** 2026-04-01

**Authors:** Albert Rosenberger, Heike Bickeböller, David C. Christiani, Neil E. Caporaso, Geoffrey Liu, Stig E. Bojesen, Loic Le Marchand, Demetrios Albanes, Melinda C. Aldrich, Adonina Tardon, Guillermo Fernández‐Tardón, Gad Rennert, John K. Field, Michael P. A. Davies, Lambertus A. Kiemeney, Philip Lazarus, Shanbeh Zienolddiny, Stephen Lam, Matthew B. Schabath, Angeline S. Andrew, Susanne M. Arnold, Gary E. Goodman, Jennifer A. Doherty, Fiona Taylor, Angela Cox, Penella J. Woll, Angela Risch, Mikael Johansson, Paul Brennan, Maria Teresa Landi, Sanjay S. Shete, Rayjean J. Hung, Christopher I. Amos, INTEGRAL‐ILCCO Consortium

**Affiliations:** 1 Department of Genetic Epidemiology, University Medical Center, GeorgAugust‐University Göttingen, Göttingen, Germany; 2 Department of Environmental Health, Harvard T.H. Chan School of Public Health and Massachusetts General Hospital, Harvard Medical School, Boston, MA USA; 3 Division of Cancer Epidemiology and Genetics, National Cancer Institute, US National Institutes of Health, Bethesda, MD USA; 4 Medical Oncology and Medical Biophysics, Princess Margaret Cancer Centre, Toronto, ON Canada; 5 Medicine and Epidemiology, Dalla Lana School of Public Health, University of Toronto, Toronto, ON Canada; 6 Department of Clinical Biochemistry, Herlev and Gentofte Hospital, Copenhagen University Hospital, Copenhagen, Denmark; 7 Faculty of Health and Medical Sciences, University of Copenhagen, Copenhagen, Denmark; 8 Copenhagen General Population Study, Herlev and Gentofte Hospital, Copenhagen, Denmark; 9 Epidemiology Program, University of Hawaii Cancer Center, Honolulu, HI USA; 10 Department of Thoracic Surgery, Division of Epidemiology, Vanderbilt University Medical Center, Nashville, TN USA; 11 Health Research Instotute of Asturias (ISPA) and University Nebrija, Asturias, Spain; 12 Clalit National Cancer Control Center at Carmel Medical Center and Technion Faculty of Medicine, Haifa, Israel; 13 Department of Molecular and Clinical Cancer Medicine, Roy Castle Lung Cancer Research Programme, The University of Liverpool, Liverpool, UK; 14 Departments of IQ Health and Urology, Radboud University Medical Center, Nijmegen, The Netherlands; 15 Department of Pharmaceutical Sciences, College of Pharmacy, Washington State University, Spokane, WA USA; 16 National Institute of Occupational Health, Oslo, Norway; 17 Department of Integrative Oncology, University of British Columbia, Vancouver, British Columbia Canada; 18 Department of Cancer Epidemiology, H. Lee Moffitt Cancer Center and Research Institute, Tampa, FL USA; 19 Department of Epidemiology, Geisel School of Medicine, Hanover, NH USA; 20 University of Kentucky Markey Cancer Center, Lexington, KY USA; 21 Swedish Medical Group, Seattle, WA USA; 22 Huntsman Cancer Institute and Department of Population Health Sciences, University of Utah, Salt Lake City, Utah, USA; 23 Department of Oncology and Metabolism, University of Sheffield, Sheffield, UK; 24 Department of Biosciences and Medical Biology, University of Salzburg and Cancer Cluster Salzburg, Salzburg, Austria; 25 Department of Radiation Sciences, Umeå University, Umeå, Sweden; 26 International Agency for Research on Cancer, World Health Organization, Lyon, France; 27 Department of Biostatistics, Division of Basic Sciences, The University of Texas MD Anderson Cancer Center, Houston, TX USA; 28 Lunenfeld-Tanenbaum Research Institute, Sinai Health System, University of Toronto, Toronto, Ontario Canada; 29 Dalla Lana School of Public Health, University of Toronto, Toronto, Canada; 30 The University of New Mexico Comprehensive Cancer Center, Albuquerque, New Mexico USA

**Keywords:** Lung cancer, Genomic marker, Rare disease, GARD, Gene-set analysis, Gene-based test, Adrenocorticotropic hormone, Hypothalamic-Pituitary-Adrenal axis, OMIM, Orphanet, CYP2A6, DMD, LTBP4

## Abstract

**Background:**

We investigated whether markers, genes or terms of the *Human Phenotype Ontology* associated with genetic or rare diseases (GARDs) that affect airway or lung function are associated with lung cancer.

**Methods:**

Genes of interest were extracted from *GARD (Genetic and Rare Diseases Information Center)*, *OMIM (*Online Mendelian Inheritance in Man®), *ORPHANET* and Monarch Initiative. Individual SNP, gene level and gene-set analyses were performed for 52,207 SNPs, 1677 genes or for 620 terms of the *Human Phenotype Ontology*. The analysis included 14,068 lung cancer cases and 12,390 cancer-free control subjects of European descent from the International Lung Cancer Consortium ILCCO.

**Results:**

The marker rs56113850 (OR=0.893, 95%CI: 0.862-0.924) was associated with lung cancer (*p*=1.2x10^-10^). This marker is located in CYP2A6 as well as in an enhancer region of *LTBP4*, which is associated with cutis laxa. A suggestive significant association was observed for two markers associated with the *DMD* gene, which is linked to Duchenne muscular dystrophy. The gene sets "Abnormal circulating adrenocorticotropin concentration" and "Central nervous system neoplasm" were found to be significantly enriched with GARD genes, and can therefore be considered to be associated with lung cancer.

**Conclusions:**

Genes associated with genetic and rare lung diseases do not generally appear to carry risk factors for lung cancer. However, genes associated with the hypothalamic-pituitary-adrenal axis show some, but rather weak or complex, associations with lung cancer. Tests at the gene level provide extremely inhomogeneous results, even when applied to the same data.

**Supplementary Information:**

The online version contains supplementary material available at 10.1186/s12885-026-15934-2.

## Introduction

Lung cancer (LC) is one of the most common and deadliest cancers worldwide. It is the second most common cancer in men and the third most common cancer in women [1]. However, LC incidence varies considerably by geographical region and sex [2]. The five-year survival rate remains low at 13–18% [3]. Smoking is the predominant but not the only known risk factor. Other important factors that increase the risk of lung cancer include exposure to harmful substances, asbestos, radon and other chemicals, as well as diseases including pneumonia, emphysema, pulmonary fibrosis and others [1].

Over the last three decades, numerous genomic loci have been identified that confer an individual genetic predisposition to LC. As part of large-scale genome-wide association studies (GWAS), individual variants have been found in genes/regions such as *CHRNA5/CHRNA3/CHRNB4* on 15q25.1, *TERT/CLPTM1L* on 5p15.33, *MSH5/BAG6* on 6p21.33, *CHEK2* on 22q12.1 and many others that co-determine susceptibility to LC [4]. The genetic landscape of susceptibility differs to some extent between Europeans and Asians, men and women, or smokers and non-smokers [5, 6]. For example, some of the strongest lung cancer susceptibility variants on 15q25, identified in Europeans, have very low allele frequencies in Asian populations, and vice versa regarding variants on 6p21.

As thousands of markers are tested in GWAS, appropriate significance levels were set to minimise spurious associations. The following definitions were proposed: 1) suggestive association, where one false-positive association is expected per GWAS, and 2) genome-wide significant association, where one false-positive association is expected to occur 0.05 times per GWAS [7]. Genome-wide significance limits range between about 1x10^−7^ and 3x10^−8^ depending on the ancestral population investigated [8]. Suggestive significance is often considered if p-values are lower than e.g 5x10^−6^ or 5×10^−4^ [9–11]. Thus with these very low significance levels of GWAS, many truely associated markers/genes remain undetected, even if the sample consists of several thousand cases and controls and the p-values are below 0.05. Some post-GWAS methods have been developed to detect genomic loci or genes associated with a trait in these grey areas [11]. E.g. complex interaction of MTAB and DKK2 were found associated with lung cancer within never smokers by gene-set (pathway-based) analyses [12]. Several methods and computer routines to perform multi-marker tests are available to enable SNP-based and gene-based association tests [13–16].

More than 7000 types of rare diseases, each affecting less than one in 2000 people, exist. Approximately 300 million people live with rare diseases. Thus their worldwide burden is significant. Around 80% of rare diseases have a genetic cause [17]. Several public available sources provide information on rare and genetic diseases, such as the *Genetic and Rare Diseases Information Center* (*GARD*, [18]) established by the US National Institutes of Health (NIH), the *OMIM database* (Online Mendelian Inheritance in Man®, [19]), the *ORPHANET* (Knowledge on rare diseases and orphan drugs, [20]) or the *Monarch Initiative* [21]. These and other data bases have been consolidated and harmonised in recent years. Data entries were further linked to the *Human Phenotype Ontology* (HPO, [22]).

Many of these rare and genetic diseases affect the respiratory system or are categorised as cancers. We therefore investigated whether markers, genes or HPO terms associated with genetic or rare diseases that affect the airways or lung function are associated with lung cancer within the large-scale series of cases and controls of European descent held by the International Lung Cancer Consortium (ILCCO)/Integrative analysis of Lung Cancer Etiology and Risk (INTEGRAL).

## Methods

The work presented has been reviewed and approved by the ILCCO Steering Committee.

### Cases and controls

We used phenotype and genotype data of 58,181 entries of the data repository of ILCCO. Details of the repository were described previously [23, 24]. DNA samples were genotyped with the Illumina Infinium OncoArray-500K. Genotype imputation was performed for all subjects in the cohort by using 32,470 reference samples from the Haplotype Reference Consortium. Individuals without information on smoking status or age, and samples of poor genotyping quality or sex discrepancies, were left out. Low-quality variants were filtered out [25]. To avoid population stratification, this analysis is focused on European-ancestry population (defined as more than 95% probability of being of European descent). 14,068 incident LC cases and 12,390 cancer-free controls of European descent remained for analysis.

### HPO classification

We retrieved all the “terms” of the *Human Phenotype Ontology* [22] and organized them according to the tree structure (levels) of the ontology. We traced and ordered each term back to its parent and grandparent and so on up to the level-1 term “HP:0000001 All”. From this, we created a list of all 979 terms from levels 2 to 6, which we reduced to 620 HPO terms of interest describing phenotypes of the respiratory system, cardiovascular system, nerves or brain, immune system including blood components, infections, inflammation or cancer.

### Selection of genes linked to genetic or rare diseases

We have brought together information of the rare diseases contained in *GARD*, *OMIM*, *ORPHANET* and *Monarch Initiative* and extracted all the genes linked to them. The data was downloaded no later than January 2023. The gene names were cross-checked against the common nomenclature of tgeo HGNC [26] to avoid ambiguities. We linked all genes with HPO terms, where HPO identifiers or linkings were provided by *GARD*, *ORPHANET* or *OMIM*. Next, 20 genes of the HLA-region, 42 genes located in regions of extended high LD, 279 immuno-related genes and 50 genes with known or more intensly investigated associations to LC were left out. We ended up with 1677 genes included in the analysis.

### Variant to gene assignment and SNP filtering

We harmonized all positions to genome reference build hg37 applying *hgLiftOver* as necessary. Next, in total 58,836 SNPs available in the investigated ILCCO sample were assigned to genes if a) located within a gene (between start codon and end codon), b) located 500bp down- and upstream of the gene to cover promoter and termination regions, or c) located in a related enhancer regions. Enhancer regions were defined according to GeneHancer 4.4. (as of 2017; gene-enhancer-score >0.5) or the EnhancerAtlas 2.0 (for tissue/cell type Lung and Bronchia_epithelial) or Super Enhancer Archive (SEA Version 3.0, Tissues: lung_fibroblasts, upper_lobe_of_left_lung, lung) Version 3.0. [27–30]. Overlapping enhancers were fused. We included markers with a missing rate <10% or p-value for HWE >0.0001 or *heterozygosity excess* between −0.30 and +0.2. We also eliminated the marker with the lower MAF of a pair of common markers (MAF>0.01 and no more than 15,000 bp apart) with D´ larger than 0.8. In addition we left out imputed SNPs with low imputation accuracy, by r^2^<50% (reduced power), Iam_HWE_<50% (genotype information is less individual-specific than population-specific) or hiQ<50% (low inter-individual heterogeneity between imputed dosages) [31–33]. We ended up with 52,207 SNPs included in the analysis.

### Statistical testing

First, we performed association tests for each marker individually using PLINK and RVTESTS, unadjusted and adjusted for 4 PCs (to infer genetic ancestry), sex and smoking status. The effect of a marker was modelled as log-additive. Since the onset of many rare and genetic diseases is at an early age, we fitted an additional model with a marker-age interaction.

In order to identify GARD genes associated with lung cancer, we performed gene-based tests compromising the whole set of markers assigned to a gene. We tested 1677 genes for multi-marker association with lung cancer, if required assuming an additive coding of genotypes. The following tests/routines were applied: permutation test (implemented in PLINK [34]); ADD-SKATO test and all SNP ADD test (implemented in REGENIE [13]); Fp test, SKATO test and Zeggini test (implemented in RVTESTS [35]).

We finally performed gene-set analyses for selected 620 HPO terms to identify those enriched with genes with low p-values for association with lung cancer, regardless of the statistical significance (*p*<0.05) of the contributing genes [36]. Kolmogorow-Smirnow tests (KS-tests) were performed. We highlighted the Leading-Edge Subset (LES) genes as the core of a gene set that accounts for the enrichment signal [37].

### Level of significance

For the purpose of this study, *p*-values were considered continuous indicators of statistical evidence. These were categorized according to the following scheme to account for multiple testing: The nominal significance level was set at $\alpha =5\%.$ The Bonferroni corrected level was given as ${\alpha }_{bon}=\alpha /k$, where k tests are performed in parallel. The suggestive level was given as ${\alpha }_{sug}=1/k$. Additionally, we considered a genome-wide level of significance of ${\alpha }_{g.w}=1/{k}_{tot}$, where ${k}_{tot}$ is the number of independent SNPs or genes in the genome.

When testing at the SNP-level, we set ${\alpha }_{g.w}^{SNP}=1\mathrm{x}{10}^{-7}$, ${\alpha }_{sug}^{SNP}=2\mathrm{x}{10}^{-6}$ (assuming k=500.000 [8]), and ${\alpha }_{bon}^{SNP}=8.5\mathrm{x}{10}^{-7}$ (given 58,836 SNPs investigated).

When testing at the gene-level, we set ${\alpha }_{g.w}^{gene}=2.58\mathrm{x}{10}^{-6}$, and ${\alpha }_{sug}^{gene}=0.0000515$ (assuming k=19.404 protein coding genes [26]), and ${\alpha }_{bon}^{gene}=0.0000298$ (given 1677 genes investigated).

When testing at the HPO-level (gene sets), we set ${\alpha }_{sug}^{HPO}=1.61x{10}^{-3}$, and ${\alpha }_{bon}^{HPO}=1.61x{10}^{-5}$ (given 620 HPO-terms investigated).

All data manipulations and analyses were performed with BCFTOOLS, VCFTOOLS, PLINK 1.90, RVTESTS, REGENIE an SAS^©^ 9.04. Links to the programs and data-sources are listed in the section Programs/URLs.

## Results

### Case-control series

The analysed series consists of 14,068 LC cases and 12,390 controls with a median age of 63. Sixty-three percent were male, 52% of cases and 28% of controls were current smokers. The most frequent histological subtype is adenocarcinoma (38%), followed by squamous cell carcinoma (26%) and small cell lung cancer (10%).

### Single SNP association

**Fig. 1:**
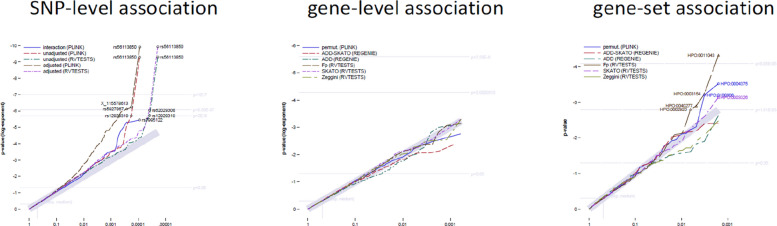
Comparative QQ-plots for the SNP-level association testing. left panel: SNP-level association testing, central panel: gene-level association testing; right panel: gene-set (HPO-term) association testing; Footnote: levels of significance are defined as *p*=0.05 (nominal) *p*=0.0000515 (suggestive) *p*=2.5810^−6^ (genome-wide); most significant markers by test with FDR<0.05 are highlighted

With a few exceptions, the p-values of PLINK and RVTESTS are sufficiently identical for the main effects (correlation of 0.99991 of the adjusted models). The corresponding QQ plot (Fig. 1, left panel) shows almost evenly distributed *p*-values across the markers, indicating a lack of significant associations.

A genome-wide significant association was observed only for rs56113850 (OR=0.893, 95%CI: 0.862–0.924.862.924, *p*=1.2x10^−10^, adjusted model), with a significant marker-age interaction (p=0.002). rs56113850 is located within the gene CYP2A6 (19q13.2).

For five other SNPs we observed suggestive significant associations. The *p*-values of these SNPs are generally low across methods, indicating robustness of the observation. Due to the selection of markers for this analysis, this low number of significant associations had to be expected.

For the Duchenne muscular dystrophy gene *DMD* (rs1545663: p=1.2x10^−6^, rs5927867: *p*=7.6x10^−7^, adjusted model), a clear role in the pathogenesis of several cancers has previously been reported [38]. In vitro functional assays revealed, the growth, migration and invasion of lung adenocarcinoma cells to be slowed when Dp71, one of the shorter dystrophin protein variants, was switched off [39]. The other suggestive significant associations related to the *ABCC6* (rs12929319: *p*=1.6x10^−5^), *NDE1* (rs62029308: *p*=1.0x10^−6^) and *SLC614* (X_115578613: *p*=5.7x10^−7^) genes remain unexplained.

### Gene-based tests

Due to the selection of genes for this analysis, we could assume an almost uniform distribution of p-values (corresponds to a normal distribution of the logit-transformed p-values around zero), presented as QQ plot in Fig. 1 (central panel). However, we observed a skewed distribution for the p-values from REGENIE, and an unusual clustering of *p*-values>0.8 to then value 1 by PLINK and RVTESTS. Therefore, all p-values were corrected by logit adjustment (logit *p*-values centred around zero),keeping their rank order of *p*-values. Details are contained in a supplement.

One to 637 genotyped markers were included in multi-marker association tests per gene. On average, 10 markers were used by PLINK, 10.5 by RVTESTS and 11.5 by REGENIE. We did not find a single gene that was at least suggestively associated with LC in a multi-marker manner with any of the methods used. All p-values of the tests at the gene level are given in the Additional file 1.

**Fig. 2:**
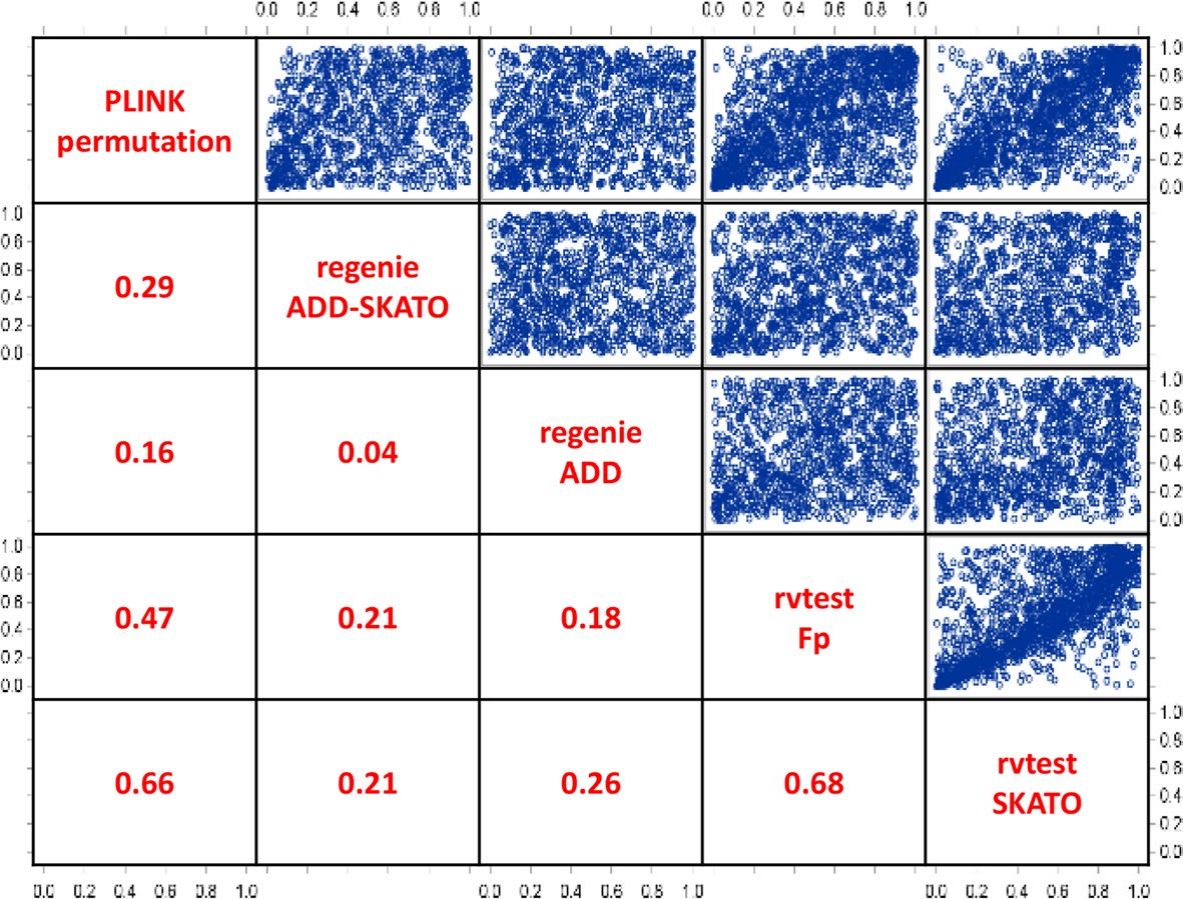
Spearman’s Correlation of p-values between methods and routines

Apart from that, the correlation of the “corrected” *p*-values between the methods and routines was found low or moderate (between r=0.04 for REGENIE-ADD vs. -ADD-SKATO and r=0.68 for RVTESTS-Fp vs. –SKATO, see Fig. 2). This indicates a non-negligible influence of the respective multi-marker association test used on the determination of "significance". This inhomogeneity is partly due to deviating marker sets that were included in the respective analyses. PLINK e.g. cannot handle imputed SNPs.

### Gene-set analyses for selected 620 HPO terms

We also had to correct the p-values of the gene-set tests by logit adjustment, but found that the difference between derived and corrected p-values was negligible. Subsequently, no routine and no test method at the gene level generally yielded lower p-values than the others. In addition, the KS test used is based on the rank order and not on the p-values themselves, and is therefore robust to skewed values. The corresponding QQ diagram (Fig. 1, right panel) displays p-values that are almost uniformly distributed across all gene sets, with a few exceptions observed when using RVTESTS-Fp.

Within the 620 gene sets tested, the HPO-term HP:0011043 “Abnormal circulating adrenocorticotropin concentration” remained significant using the *p*-values of Fp-statistics after Bonferroni correction (p_corr._=4.7 x10^−5^, p_uncorr._=4.3x10^−5^, RVTESTS-Fp). Only four of the 23 leading-edge genes (GMPPA, CYP11B1, POR, SMO) are themselves at least nominally significant (*p*<0.05). When using results from other gene-level tests, the *p*-values of this HPO-term ranged from 0.003 (RVTESTS -SKATO and -Zeggini) to 0.18 (REGENIE-ADD-SKATO) Table 1.

Suggestively significant enrichment of associated genes was observed for six further HPO-terms (4x based on *p*-values of RVTESTS, 2x based on PLINK results). Most *p*-values of these HPO-terms, when using results from other gene-level tests, were generally low (<0.25). We found 111 leading-edge genes in total over all seven HPO-terms (listed in Supplement S-Table 1). For 12 of them, the gene-level *p*-value were <0.05, as determined by at least one of the applied tests (see Table 2). These 12 top genes can be divided into three categories of HPO terms. First, *BDNF, WDPCP, APC* and SMO are associated with neoplasms of the nervous system, especially the pituitary gland (group BN). Second, APC, ENO3, ACAD9, RNASEH1, FKTN, DYSF, and ATP2A1 are associated with Myalgia. Third, CYP11B1 and POR (other cytochrome P450 enzymes), GMPPA, and SMO are associated with the adrenocorticotropin (group ACTH).

**Table 1:** At least suggestively significant HPO terms

**HPO class**	**HPO no.**	**test applied**	**p**_**corr**_^**†**^	**# LE genes (# p<0.05)**	**HPO term**
	**Bonferroni correction:**${\boldsymbol{p}}\le 1.61{\boldsymbol{x}}{10}^{-5}$				
ACTH	HPO:0011043	RVTESTS -Fp	4.7 x10^−5^	23 (4)	Abnormal circulating ACTH concentration
	**Suggestive significance:**${\boldsymbol{p}}\le 1.61{\boldsymbol{x}}{10}^{-2}$				
BN	HPO:0004375	PLINK-permutation	0.0003	53 (3)	Neoplasm of the nervous system
ACTH	HPO:0003154	RVTESTS-Fp	0.0006	13 (3)	Increased circulating ACTH level
BN	HPO:0100006	PLINK-permutation	0.0006	44 (3)	Neoplasm of the central nervous system
Myalgia	HPO:0003326	RVTESTS-SKATO	0.0007	57 (7)	Myalgia
BN	HPO:0040277	RVTESTS -Fp	0.0010	25 (2)	Neoplasm of the pituitary gland
ACTH	HPO:0002920	RVTESTS -Fp	0.0020	12 (1)	Decreased circulating ACTH concentration

p_corr_^†^ corrected p-values of a Kolmogorow-Smirnow test for gene-sets; ^ŧ^ *p*-value according to the listed applied test; *ACTH* adrenocorticotropin hormone, *BN* brain/nervours system, *LE* leading-edge, *HPO* Human Phenotype Ontology

**Table 2:** Significant (pmin<0.05) LE genes in at least suggestively significant HPO terms

**GENE**	**Minimal *P*-Value**^**†**^	**Test**	**Class 1**	**2**	**3**	**4/5**	**HPO no.**	**-**	**-**	**-**	**-**
*BDNF*	0.03	RVTESTS-Zeggini	BN	BN			HPO:0004375	HPO:0100006			
*MAPT*	0.02	RVTESTS-SKATO	BN	BN			HPO:0004375	HPO:0100006			
*APC*	0.005	RVTESTS-Fp	Myalgia	BN	BN	BN	HPO:0003326	HPO:0004375	HPO:0040277	HPO:0100006	
*SMO*	0.02	RVTESTS-Fp	ACTH	BN	ACTH	BN	HPO:0002920	HPO:0004375	HPO:0011043	HPO:0040277	HPO:0100006
*BRAF*	0.02	RVTESTS-Zeggini	ACTH	ACTH	BN		HPO:0003154	HPO:0011043	HPO:0040277		
*CYP11B1*	0.005	PLINK-permutation	ACTH	ACTH			HPO:0003154	HPO:0011043			
*GMPPA*	0.01	RVTESTS-Fp	ACTH	ACTH			HPO:0003154	HPO:0011043			
*POR*	0.02	RVTESTS-Fp	ACTH	ACTH			HPO:0003154	HPO:0011043			
*ENO3*	0.003	RVTESTS-Zeggini	Myalgia				HPO:0003326				
*LPIN2*	0.01	REGENIE-ADD	Myalgia				HPO:0003326				
*ACAD9*	0.02	RVTESTS-SKATO	Myalgia				HPO:0003326				
*FKTN*	0.02	RVTESTS-Fp	Myalgia				HPO:0003326				
*RNASEH1*	0.02	RVTESTS-SKATO	Myalgia				HPO:0003326				
*ACP5*	0.03	REGENIE-SKATO	Myalgia				HPO:0003326				
*DMD*	0.03	PLINK-permutation	Myalgia				HPO:0003326				
*PTPN2*	0.03	RVTESTS-Fp	Myalgia				HPO:0003326				
*ATP2A1*	0.04	PLINK-permutation	Myalgia				HPO:0003326				
*CAPN3*	0.04	REGENIE-ADD	Myalgia				HPO:0003326				
*DYSF*	0.04	RVTESTS-SKATO	Myalgia				HPO:0003326				

^†^ minimal *p*-value of all gene-level tests applied; *HPO* Human Phenotype Ontology

### Functional gene network

Because mapping the interactions of proteins coded by associated genes is a key to understanding complex cellular mechanisms, we used *funcoup 6* [40, 41] to build human tissue-specific networks of all 111 leading-edge genes of the at least suggestively significant HPO terms. Multiple multi-omics data, collected from public online databases and other sources (none of which were compiled by ILCCO), are integrated with machine learning techniques by *funcoup 6* to represent interactomes. The tissue specificity for humans is based on protein expression extracted from Human Protein Atlas [42]. The networks only contain genes with an expression greater than zero, but are not limited to the leading-edge genes we specified in advance.. We restricted the construction to the following tissues: lung, bronchus, nasopharynx, lymph nodes cerebral cortex, cerebellum, and hippocampus.

**Fig. 3:**
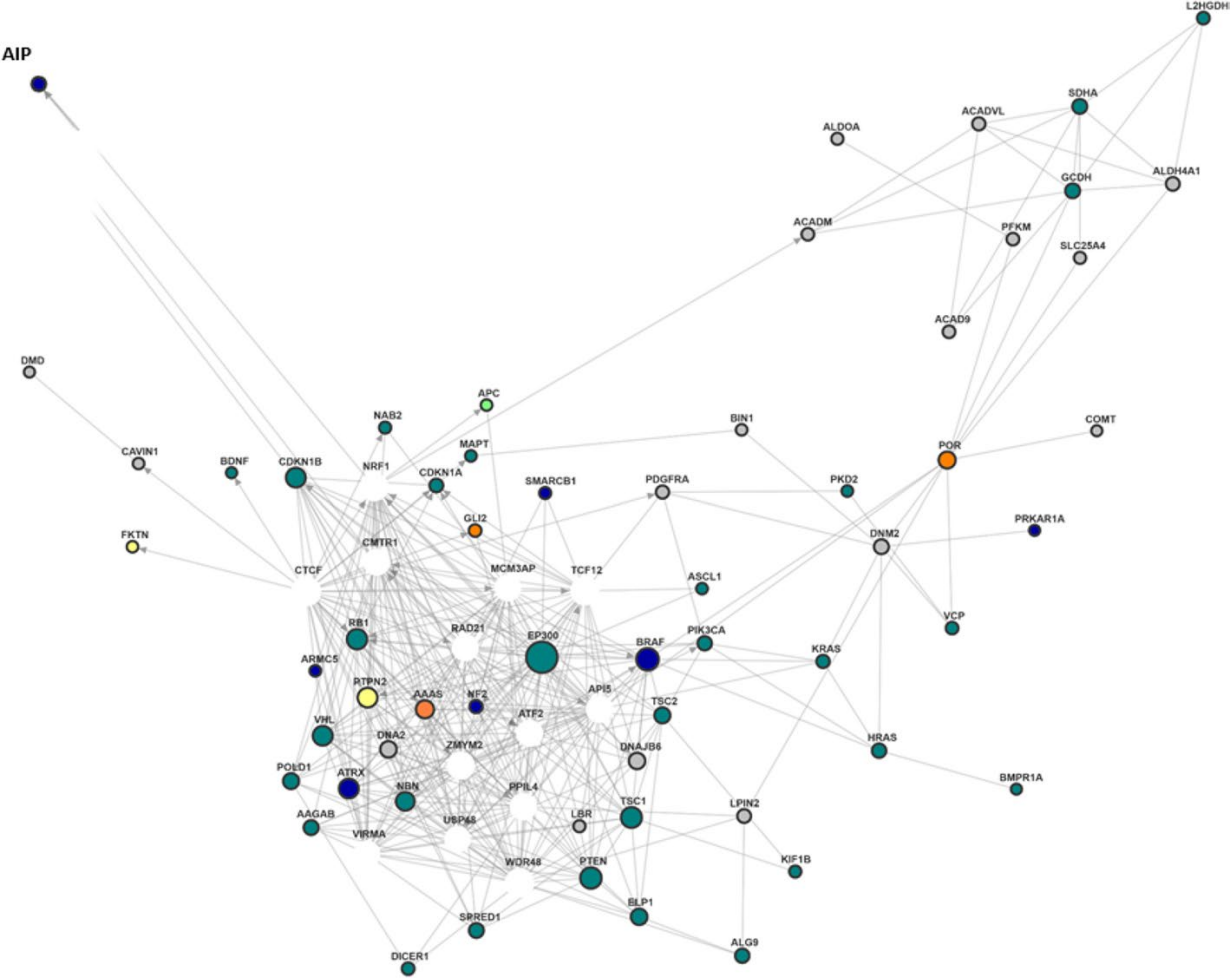
Gene expression network of LE-genes of at least suggestive HPO-terms. Gene expression network created by funcoup 6; only leading-edge genes of at least suggestive significant HPO-term are displayed. Green dots: genes related to nervous system/pituitary gland. Orange dots: genes related to ACTH circulation/level. Blue dots: genes related to both the nervous system/pituitary gland and the ACTH cycle/concentration. Grey dots: genes related to Myalgia. Light/yellow dots: gene-level *p*-value<0.05; white dots: added by funcoup 6 for building the network

Although the list of leading-edge genes was compiled based on the association of DNA variants with LC, a compact but complex network of gene expressions was found (Fig. 3). The core of the network is mainly made up from leading-edge genes related to HPO-class BN (nervous system/pituitary gland; green dots). However, central in this core net one can find the genes *NF2* and *BRAF* (associated to HPO-class BN and ACTH), *EP300* (associated to HPO-class BN), *AAAS* (associated to HPO-class ACTH) and *PTPN2* (associated to Myalgia). The single gene-level p-values of these genes range from 0.03 to 0.27 (RVTESTS-Fp).

There is also a separate subnetwork that is only connected to the core net via the *POR* gene (cytochrome P450 oxidoreductase), a leading-edge gene of the association to Myalgia. Interestingly, the *POR* variation 503 V was associated with faster *CYP2A6* activity (higher nicotine metabolite ratio) [43]. Its single gene-level *p*-value was 0.02 (RVTESTS-Fp). Only a few genes, such as *AIP* or *DMD* (discussed before), can be regarded as satellites outside the core network.

## Discussion

The ILCCO has compiled a large-scale genotype dataset of thousands of lung cancer cases and control subjects in a joint effort of several studies. Using this dataset, numerous genomic loci associated with lung cancer risk have been detected. However, it can also be assumed that other, as yet undiscovered, genes are associated with lung cancer, especially if they are involved in complex molecular structures. We aimed to discover such genes and structures by focusing on rare diseases that affect the respiratory system.

By chance, we are able to re-detect the single-marker association of the marker rs56113850, which is located in the gene *CYP2A6* (19q13.2). This marker has already been associated to lung cancer in a joint GWAS of the ILCCO [24] and is considered primarily informative for LC prediction [12]. Therefore, rs56113850 was initially left out from this analysis but included again in the group of analysed markers as it is located in an enhancer region for the GARD gene LTBP4 (e.g. associated with cutis laxa with severe lung abnormalities, ORPHA code 221145, *OMIM* no. 613177). In most smokers, *CYP2A6*-catalyzed C-oxidation accounts for >75% of nicotine metabolism. The activity of this major nicotine-metabolizing enzyme has been shown to correlate with the amount of nicotine and carcinogens drawn from cigarettes [43, 44]. *CYP2A6* polymorphisms are well known to alter tobacco-related cancer risks [45, 46]. Smoking intensity accounted for 82.3% of the effect of *CYP2A6* activity on lung cancer risk but entirely mediated the genetic effect of rs56113850 [47].

However, the pleiotropy of *CYP2A6* and *LTBP4* may be the molecular reason why tobacco smoking can lead to a rapid, severe loss of lung function in individuals with *LTBP4*-related cutis laxa [48, 49].

Two further markers (rs1545663, rs5927867) are associated with LC with suggestive significance. These can be assigned to the Duchenne muscular dystrophy gene *DMD*. A clear role in the pathogenesis of various types of cancer has been reported for this gene [38]. Furthermore, the growth, migration and invasion of lung adenocarcinoma cells were slowed when Dp71, one of the shorter dystrophin protein variants, was switched off in in vitro functional assays [39].

Although being unable to prove any single GARD gene to be associated with LC, a statistically significant enrichment of genes involved in an abnormal circulating adrenocorticotropic concentration (three HPO-Terms) was perceived. The adrenocorticotropic hormone (ACTH) is mainly produced by the anterior pituitary gland. Via the Hypothalamic-Pituitary-Adrenal (HPA) axis, it stimulates the production and release of cortisol. The HPA system hence influences the central nervous system and the endocrine system by regulating the balance of hormones in response to stress [50]. Tumours outside the pituitary gland are known to also produce ACTH; in particular, a large proportion of SCLC (around 85–90%) expresses neuroendocrine biomarkers such as adrenocorticotropic hormone (ACTH) [51].

It is reported that nicotine is a strong activator of the hypothalamus pituitary adrenal (HPA) axis [52, 53]. Smoking of only two cigarettes is said to consistently activate the HPA axis of habitual smokers. On the other hand, chronic inflammation of the airways is a common consequence of habitual smoking, and smokers often present with low-grade systemic inflammation, which may be mediated by HPA axis alterations. The main effective component of cigarette smoke on the HPA axis seems to be nicotine [54]. Therefore, it does not seem surprising that some genes that are more or less directly involved in the regulation of the HPA axis show a weak and barely discernible association with lung cancer. We could not identify an explicit functional pathway that should be considered relevant for lung cancer risk. However, we found that most of the leading genes of our analysis that seem to be somehow connected with the HPA axis interact in a complex manner. Finally, however, we must adhere that it seems unlikely that such weak or entangled associations can make a useful contribution to risk assessment with regard to lung cancer.

Numerous region- and gene-based multi-marker aggregation tests have been proposed to overcome the challenge of analysing rare variants, among other things [55]. We applied five of these, provided by PLINK, RVTESTS or REGENIE. The resulting p-values were extremely inhomogeneous, even though the input-data for each test was the same. The tests used were based on very different methods for combining multi-marker information into a single test statistic. Burden tests (RVTESTS: Zeggini and Fp, REGENIE: ADD) aggregate all genotypes into a kind of summary dose. They are considered powerful when a large proportion of variants are causal and effects are in the same direction (e.g. all rare alleles are the risk alleles) [55]. Different weighting schemes may heavily influence gene-level *p*-values [56]. In contrast, variance-component tests evaluate the distribution of genetic effects for a group of markers. This is powerful if the direction of the effects differ or the fraction of causal SNPs is low. A robust middle ground is achieved through e.g. a Cauchy combination of such test procedures (RVTESTS: SKAT-O; REGENIE: ADD-SKAT-O). In contrast, PLINK's permutation test average chi-square test statistics of only a subset of the most significant variants. *P*-values are determined by phenotype-permutation [57]. Hence, common markers are usually more likely to contribute to the gene-level *p*-values than rare markers. Since the actual underlying genetic architectures can vary between the considered genes and, moreover, are all unknown, no method or routine could be preferred a priori. It is therefore all the more remarkable to see how strongly the *p*-values appear to be influenced by the method, in this real-data application. Without prior knowledge, the results of the multi-marker tests seem almost diced. Therefore, we have little confidence in the validity of these tests.

## Conclusions

Genes associated with genetic and rare lung diseases do not appear to be risk factors for lung cancer. However, genes related to the hypothalamic-pituitary-adrenal (HPA) axis show some, but rather weak or intricate associations with lung cancer. Tests at the gene level provide extremely inhomogeneous results, even when applied to the same data.

## Supplementary Information

Supplementary Material 1.

Supplementary Material 2.

## Data Availability

The case-control data that support the findings of this study are available from ILCCO/INTEGRAL from the authors upon reasonable request and with permission from the ILCCO/INTEGRAL data access committee. The following publicly available datasets were used in this work: Prostate, Lung, Colorectal, and Ovarian Cancer Screening Trial, phs000093.v2.p2; FLCCA study, phs000716.v1.p1; EAGLE study, phs000336.v1.p1; German, SLRI, IARC, and MD Anderson Cancer Center studies, phs000876.v2.p1; Oncoarray study, phs001273.v3.p2; imputed Oncoarray study using HRC reference panel, phs001273.v4.p2;

## References

[1] Siegel RL, Kratzer TB, Giaquinto AN, Sung H, Jemal A. Cancer statistics, 2025. CA: A Cancer Journal for Clinicians. 2025;75:10–45. 10.3322/caac.21871. 39817679 10.3322/caac.21871 PMC11745215

[2] Zhang Y, Vaccarella S, Morgan E, Li M, Etxeberria J, Chokunonga E, et al. Global variations in lung cancer incidence by histological subtype in 2020: a population-based study. Lancet Oncol. 2023;24:1206–18. 10.1016/S1470-2045(23)00444-8. 37837979 10.1016/S1470-2045(23)00444-8

[3] De Angelis R, Sant M, Coleman MP, Francisci S, Baili P, Pierannunzio D, et al. Cancer survival in Europe 1999-2007 by country and age: results of EUROCARE--5-a population-based study. Lancet Oncol. 2014;15:23–34. 10.1016/S1470-2045(13)70546-1. 24314615 10.1016/S1470-2045(13)70546-1

[4] Bosse Y, Amos CI. A decade of GWAS results in lung cancer. Cancer Epidemiol Biomarkers Prev. 2018;27:363–79. 10.1158/1055-9965.EPI-16-0794. 28615365 10.1158/1055-9965.EPI-16-0794 PMC6464125

[5] Shi J, Shiraishi K, Choi J, Matsuo K, Chen T-Y, Dai J, et al. Genome-wide association study of lung adenocarcinoma in East Asia and comparison with a European population. Nat Commun. 2023;14:3043. 10.1038/s41467-023-38196-z. 37236969 10.1038/s41467-023-38196-z PMC10220065

[6] Gorman BR, Ji S-G, Francis M, Sendamarai AK, Shi Y, Devineni P, et al. Multi-ancestry GWAS meta-analyses of lung cancer reveal susceptibility loci and elucidate smoking-independent genetic risk. Nat Commun. 2024;15:8629. 10.1038/s41467-024-52129-4. 39366959 10.1038/s41467-024-52129-4 PMC11452618

[7] Duggal P, Gillanders EM, Holmes TN, Bailey-Wilson JE. Establishing an adjusted p-value threshold to control the family-wide type 1 error in genome wide association studies. BMC Genomics. 2008;9:516. 18976480 10.1186/1471-2164-9-516 PMC2621212

[8] Kanai M, Tanaka T, Okada Y. Empirical estimation of genome-wide significance thresholds based on the 1000 Genomes Project data set. J Hum Genet. 2016;61:861–6. 10.1038/jhg.2016.72. 27305981 10.1038/jhg.2016.72 PMC5090169

[9] Burton PR, Clayton DG, Cardon LR, Craddock N, Deloukas P, Duncanson A, et al. Genome-wide association study of 14,000 cases of seven common diseases and 3,000 shared controls. Nature. 2007;447:661–78. 10.1038/nature05911. 17554300 10.1038/nature05911 PMC2719288

[10] Hammond RK, Pahl MC, Su C, Cousminer DL, Leonard ME, Lu S, et al. Biological constraints on GWAS SNPs at suggestive significance thresholds reveal additional BMI loci. Elife. 2021;10:e62206. 10.7554/eLife.62206. 33459256 10.7554/eLife.62206 PMC7815306

[11] Adam Y, Samtal C, Brandenburg J, Falola O, Adebiyi E. Performing post-genome-wide association study analysis: overview, challenges and recommendations. F1000Res. 2021;10:1002. 10.12688/f1000research.53962.1. 35222990 10.12688/f1000research.53962.1 PMC8847724

[12] Rosenberger A, Muttray N, Hung RJ, Christiani DC, Caporaso NE, Liu G, et al. Gene–gene interaction of AhRwith and within the Wntcascade affects susceptibility to lung cancer. Eur J Med Res. 2022;27:14. 10.1186/s40001-022-00638-7. 35101137 10.1186/s40001-022-00638-7 PMC8805279

[13] Mbatchou J, Barnard L, Backman J, Marcketta A, Kosmicki JA, Ziyatdinov A, et al. Computationally efficient whole-genome regression for quantitative and binary traits. Nat Genet. 2021;53:1097–103. 10.1038/s41588-021-00870-7. 34017140 10.1038/s41588-021-00870-7

[14] Ionita-Laza I, Lee S, Makarov V, Buxbaum JD, Lin X. Sequence kernel association tests for the combined effect of rare and common variants. Am J Hum Genet. 2013;92:841–53. 10.1016/j.ajhg.2013.04.015. 23684009 10.1016/j.ajhg.2013.04.015 PMC3675243

[15] Li B, Leal SM. Methods for detecting associations with rare variants for common diseases: application to analysis of sequence data. Am J Hum Genet. 2008;83:311–21. 10.1016/j.ajhg.2008.06.024. 18691683 10.1016/j.ajhg.2008.06.024 PMC2842185

[16] Lee S, Wu MC, Lin X. Optimal tests for rare variant effects in sequencing association studies | Biostatistics | Oxford academic. Biostatistics. 2012;13:762–75. 10.1093/biostatistics/kxs014. 22699862 10.1093/biostatistics/kxs014 PMC3440237

[17] Health TLG. The landscape for rare diseases in 2024. The Lancet Global Health. 2024;12:e341. 10.1016/S2214-109X(24)00056-1. 38365397 10.1016/S2214-109X(24)00056-1

[18] Zhu Q, Nguyen D-T, Grishagin I, Southall N, Sid E, Pariser A. An integrative knowledge graph for rare diseases, derived from the Genetic and Rare Diseases Information Center (GARD). J Biomed Semantics. 2020;11:13. 10.1186/s13326-020-00232-y. 33183351 10.1186/s13326-020-00232-y PMC7663894

[19] Amberger JS, Hamosh A. Searching Online Mendelian Inheritance in Man (OMIM): A Knowledgebase of Human Genes and Genetic Phenotypes. Curr Protocols Bioinformatics. 2017;58:1 2 1-1 2 12. 10.1002/cpbi.27. 10.1002/cpbi.27 PMC5662200 28654725

[20] INSERM US14. Orphanet. http://www.orpha.net/consor/www/cgi-bin/index.php?lng=DE. Accessed 16 Dec 2022.

[21] Mungall CJ, McMurry JA, Kohler S, Balhoff JP, Borromeo C, Brush M, et al. The Monarch Initiative: an integrative data and analytic platform connecting phenotypes to genotypes across species. Nucleic Acids Res. 2017;45:D712-22. 10.1093/nar/gkw1128. 27899636 10.1093/nar/gkw1128 PMC5210586

[22] Gargano MA, Matentzoglu N, Coleman B, Addo-Lartey EB, Anagnostopoulos AV, Anderton J, et al. The Human Phenotype Ontology in 2024: phenotypes around the world. Nucleic Acids Res. 2024;52:D1333-46. 10.1093/nar/gkad1005. 37953324 10.1093/nar/gkad1005 PMC10767975

[23] Amos CI, Dennis J, Wang Z, Byun J, Schumacher FR, Gayther SA, et al. The OncoArray Consortium: a network for understanding the genetic architecture of common cancers. Cancer Epidemiol Biomarkers Prev. 2017;26:126–35. 10.1158/1055-9965.EPI-16-0106. 27697780 10.1158/1055-9965.EPI-16-0106 PMC5224974

[24] McKay JD, Hung RJ, Han Y, Zong X, Carreras-Torres R, Christiani DC, et al. Large-scale association analysis identifies new lung cancer susceptibility loci and heterogeneity in genetic susceptibility across histological subtypes. Nat Genet. 2017;49:1126–32. 10.1038/ng.3892. 28604730 10.1038/ng.3892 PMC5510465

[25] Cheng C, Hong W, Li Y, Xiao X, McKay J, Han Y, et al. Mosaic chromosomal alterations are associated with increased lung cancer risk: insight from the INTEGRAL-ILCCO cohort analysis. J Thorac Oncol. 2023;18:1003–16. 10.1016/j.jtho.2023.05.001. 37150255 10.1016/j.jtho.2023.05.001 PMC10435278

[26] Gray KA, Yates B, Seal RL, Wright MW, Bruford EA. Genenames.org: the HGNC resources in 2015. Nucleic Acids Res. 2015;43(Database issue):D1079-85. 10.1093/nar/gku1071. 25361968 10.1093/nar/gku1071 PMC4383909

[27] Fishilevich S, Nudel R, Rappaport N, Hadar R, Plaschkes I, Iny Stein T, et al. GeneHancer: genome-wide integration of enhancers and target genes in GeneCards. Database (Oxford). 2017;2017:bax028. 10.1093/database/bax028. 28605766 10.1093/database/bax028 PMC5467550

[28] Ma S, Dalgleish J, Lee J, Wang C, Liu L, Gill R, et al. Powerful gene-based testing by integrating long-range chromatin interactions and knockoff genotypes. Proc Natl Acad Sci U S A. 2021;118:e2105191118. 10.1073/pnas.2105191118. 34799441 10.1073/pnas.2105191118 PMC8617518

[29] Li X, Li Z, Zhou H, Gaynor SM, Liu Y, Chen H, et al. Dynamic incorporation of multiple in silico functional annotations empowers rare variant association analysis of large whole genome sequencing studies at scale. Nat Genet. 2020;52:969. 10.1038/s41588-020-0676-4. 32839606 10.1038/s41588-020-0676-4 PMC7483769

[30] Gao T, Qian J. EnhancerAtlas 2.0: an updated resource with enhancer annotation in 586 tissue/cell types across nine species. Nucleic Acids Research. 2020;48:D58-64. 10.1093/nar/gkz980. 31740966 10.1093/nar/gkz980 PMC7145677

[31] Rosenberger A, Tozzi V, Bickeböller H, Hung RJ, Christiani DC, Caporaso NE, et al. Iam hiQ—a novel pair of accuracy indices for imputed genotypes. BMC Bioinform. 2022;23:50. 10.1186/s12859-022-04568-3. 10.1186/s12859-022-04568-3 PMC8785528 35073846

[32] Thormann KA, Tozzi V, Starke P, Bickeböller H, Baum M, Rosenberger A. ImputAccur: fast and user-friendly calculation of genotype-imputation accuracy-measures. BMC Bioinform. 2022;23:316. 10.1186/s12859-022-04863-z. 10.1186/s12859-022-04863-z PMC9351229 35927623

[33] Marchini J, Howie B. Genotype imputation for genome-wide association studies. Nat Rev Genet. 2010;11:499–511. 10.1038/nrg2796. 20517342 10.1038/nrg2796

[34] Chang CC, Chow CC, Tellier LC, Vattikuti S, Purcell SM, Lee JJ. Second-generation PLINK: rising to the challenge of larger and richer datasets. Gigascience. 2015;4:1–16. 10.1186/s13742-015-0047-8. 25722852 10.1186/s13742-015-0047-8 PMC4342193

[35] Zhan X, Hu Y, Li B, Abecasis GR, Liu DJ. RVTESTS: an efficient and comprehensive tool for rare variant association analysis using sequence data. Bioinformatics. 2016;32:1423–6. 10.1093/bioinformatics/btw079. 27153000 10.1093/bioinformatics/btw079 PMC4848408

[36] Maciejewski H. Gene set analysis methods: statistical models and methodological differences. Brief Bioinform. 2014;15:504–18. 10.1093/bib/bbt002. 23413432 10.1093/bib/bbt002 PMC4103537

[37] Subramanian A, Tamayo P, Mootha VK, Mukherjee S, Ebert BL, Gillette MA, et al. Gene set enrichment analysis: a knowledge-based approach for interpreting genome-wide expression profiles. Proc Natl Acad Sci U S A. 2005;102:15545–50. 16199517 10.1073/pnas.0506580102 PMC1239896

[38] Jones L, Naidoo M, Machado LR, Anthony K. The Duchenne muscular dystrophy gene and cancer. Cell Oncol (Dordr). 2021;44:19–32. 10.1007/s13402-020-00572-y. 33188621 10.1007/s13402-020-00572-y PMC7906933

[39] Tan S, Tan Sipin, Chen Zhikang, Cheng Ke, Chen Zhicao, Wang Wenmei, et al. Knocking down Dp71 expression in A549 cells reduces its malignancy in vivo and in vitro. Cancer Investigation. 2016;34:16–25. 10.3109/07357907.2015.1084002. 26691328 10.3109/07357907.2015.1084002

[40] Buzzao D, Persson E, Guala D, Sonnhammer ELL. FunCoup 6: advancing functional association networks across species with directed links and improved user experience. Nucleic Acids Res. 2025;53:D658-71. 10.1093/nar/gkae1021. 39530220 10.1093/nar/gkae1021 PMC11701656

[41] Persson E, Castresana-Aguirre M, Buzzao D, Guala D, Sonnhammer ELL. FunCoup 5: Functional Association Networks in All Domains of Life, Supporting Directed Links and Tissue-Specificity. J Mol Biol. 2021;433:166835. 10.1016/j.jmb.2021.166835. 33539890 10.1016/j.jmb.2021.166835

[42] The Human Protein Atlas. https://www.proteinatlas.org/. Accessed 5 Feb 2021.

[43] Chenoweth MJ, Zhu AZX, Sanderson Cox L, Ahluwalia JS, Benowitz NL, Tyndale RF. Variation in P450 oxidoreductase (POR) A503V and flavin-containing monooxygenase (FMO)-3 E158K is associated with minor alterations in nicotine metabolism, but does not alter cigarette consumption. Pharmacogenet Genomics. 2014;24:172–6. 10.1097/FPC.0000000000000031. 24448396 10.1097/FPC.0000000000000031 PMC3985268

[44] Park SL, Murphy SE, Wilkens LR, Stram DO, Hecht SS, Le Marchand L. Association of CYP2A6 activity with lung cancer incidence in smokers: The multiethnic cohort study. PLoS One. 2017;12:e0178435. 10.1371/journal.pone.0178435. 28542511 10.1371/journal.pone.0178435 PMC5444837

[45] Wang L, Zang W, Liu J, Xie D, Ji W, Pan Y. Association of CYP2A6*4 with susceptibility of lung cancer: a meta-analysis. PLoS One. 2013;8:e59556. 10.1371/journal.pone.0059556. 23585826 10.1371/journal.pone.0059556 PMC3622000

[46] Johani FH, Majid MSA, Azme MH, Nawi AM. Cytochrome P450 2A6 whole-gene deletion ( CYP2A6*4 ) polymorphism reduces risk of lung cancer: A meta-analysis. Tob Induc Dis. 2020;18 June. 10.18332/tid/122465. 10.18332/tid/122465 PMC7291960 32547353

[47] Du M, Xin J, Zheng R, Yuan Q, Wang Z, Liu H, et al. CYP2A6 Activity and Cigarette Consumption Interact in Smoking-Related Lung Cancer Susceptibility. Cancer Res. 2024;84:616–25. 10.1158/0008-5472.can-23-0900. 38117513 10.1158/0008-5472.CAN-23-0900 PMC11184964

[48] Callewaert BL, Urban Z. LTBP4-Related Cutis Lax. 1993. 26866239

[49] Corbett E, Glaisyer H, Chan C, Madden B, Khaghani A, Yacoub M. Congenital cutis laxa with a dominant inheritance and early onset emphysema. Thorax. 1994;49:836–7. 10.1136/thx.49.8.836. 8091333 10.1136/thx.49.8.836 PMC475135

[50] Oh I-J, Kim K-S, Kim Y-C, Park J-Y, Yoo K-Y, Do S-H, et al. Altered Hypothalamus-Pituitary-Adrenal Axis Function: A Potential Underlying Biological Pathway for Multiple Concurrent Symptoms in Patients With Advanced Lung Cancer. Psychosom Med. 2019;81:41. 10.1097/PSY.0000000000000648. 30371632 10.1097/PSY.0000000000000648

[51] Pandjarova I, Mercieca D, Gijtenbeek RGP, Pereira JO, Fantin A, Castaldo N, et al. Small cell lung cancer and neuroendocrine tumours. Breathe. 2024;20:240004. 10.1183/20734735.0004-2024. 39534494 10.1183/20734735.0004-2024 PMC11555584

[52] Rohleder N, Kirschbaum C. The hypothalamic-pituitary-adrenal (HPA) axis in habitual smokers. Int J Psychophysiol. 2006;59:236–43. 10.1016/j.ijpsycho.2005.10.012. 16325948 10.1016/j.ijpsycho.2005.10.012

[53] LaFond M, DeAngelis B, al’Absi M. Hypothalamic pituitary adrenal and autonomic nervous system biomarkers of stress and tobacco relapse: Review of the research. Biol Psychol. 2024;192:108854. 10.1016/j.biopsycho.2024.108854. 39151748 10.1016/j.biopsycho.2024.108854

[54] Rohleder N, Kirschbaum C. The hypothalamic–pituitary–adrenal (HPA) axis in habitual smokers. Int J Psychophysiol. 2006;59:236–43. 10.1016/j.ijpsycho.2005.10.012. 16325948 10.1016/j.ijpsycho.2005.10.012

[55] Lee S, Abecasis GR, Boehnke M, Lin X. Rare-variant association analysis: study designs and statistical tests. Am J Hum Genet. 2014;95:5–23. 10.1016/j.ajhg.2014.06.009. 24995866 10.1016/j.ajhg.2014.06.009 PMC4085641

[56] Povysil G, Petrovski S, Hostyk J, Aggarwal V, Allen AS, Goldstein DB. Rare-variant collapsing analyses for complex traits: guidelines and applications. Nat Rev Genet. 2019;20:747–59. 10.1038/s41576-019-0177-4. 31605095 10.1038/s41576-019-0177-4

[57] Chang C. PLINK - Association analysis. 2026. https://www.cog-genomics.org/plink/1.9/assoc#set. Accessed 25 Aug 2025.

