# Supplementary material for: Genes associated with genetic and rare lung diseases and the risk of lung cancer
Source: BMC Cancer. 2026 Apr 1;26:461. doi: 10.1186/s12885-026-15934-2 (PMC13063928; doi:10.1186/s12885-026-15934-2)
Supplement: Supplementary file 1 — Supplementary Material 1. [file 12885_2026_15934_MOESM1_ESM.pdf]

Supplementary Material

Logit adjustment of p-values

The left upper panel of S-Figure 1 displays the distribution of p-value from gene-based test. The left upper panel of S-Figure 1 displays the corresponding logit-transformed values: $x = \ln(\frac{p}{1-p})$, per method and routine. The deviation from the expected uniform distribution is clearly recognizable.

The mean m* and the standard deviation s* of equally distributed values (theoretical distribution) in the space 0-1 were determined.

The logit-transformed observed p-values were standardized to m* and s*. The standardized logit values were finally converted back into “corrected” p-values.

The left lower panel of S-Figure 1 displays the standardized logit values, per method and routine. The left lower panel of S-Figure 1 displays the distribution of “corrected” p-value from gene-based test, now following a uniform distribution.

S-Figure 2 displays the same for gene-set association testing.

S-Figure 1 Distribution on p-/logit-values before and after correction: gene-level association testing

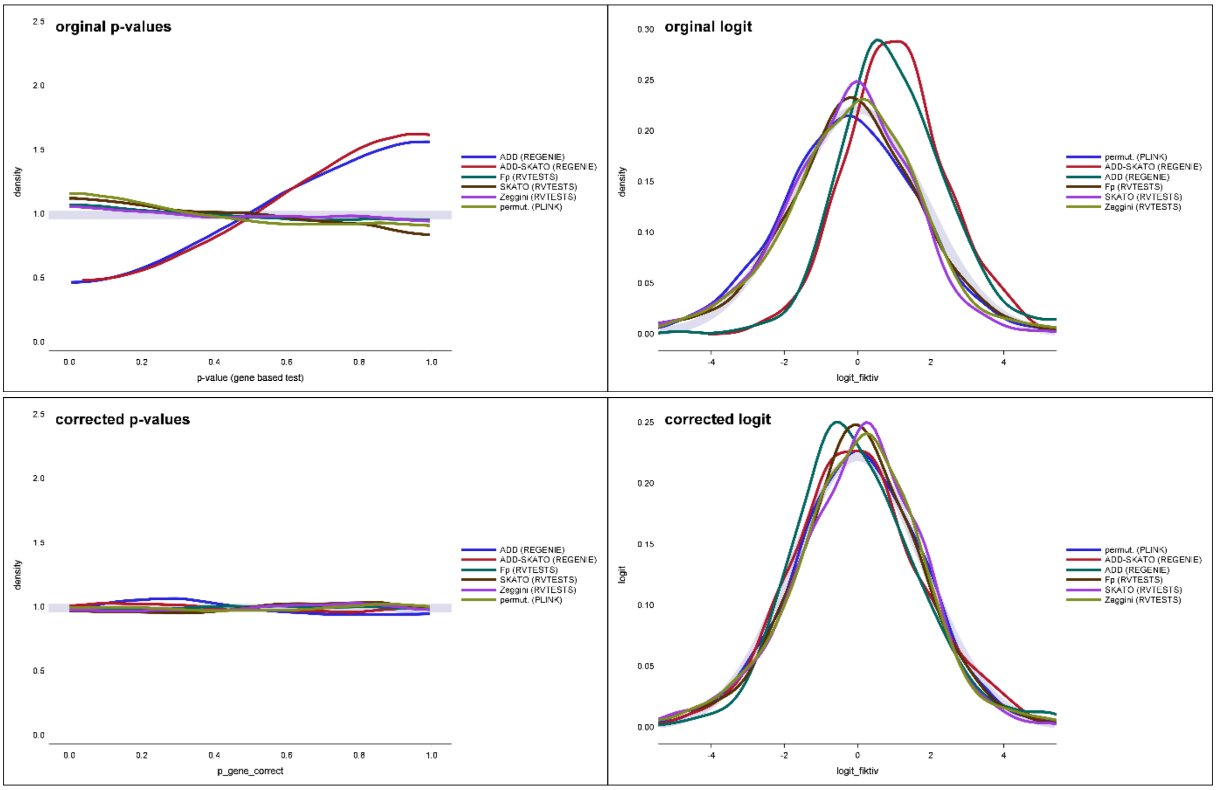

S-Figure 2 Distribution on p-/logit-values before and after correction: gene-set association testing

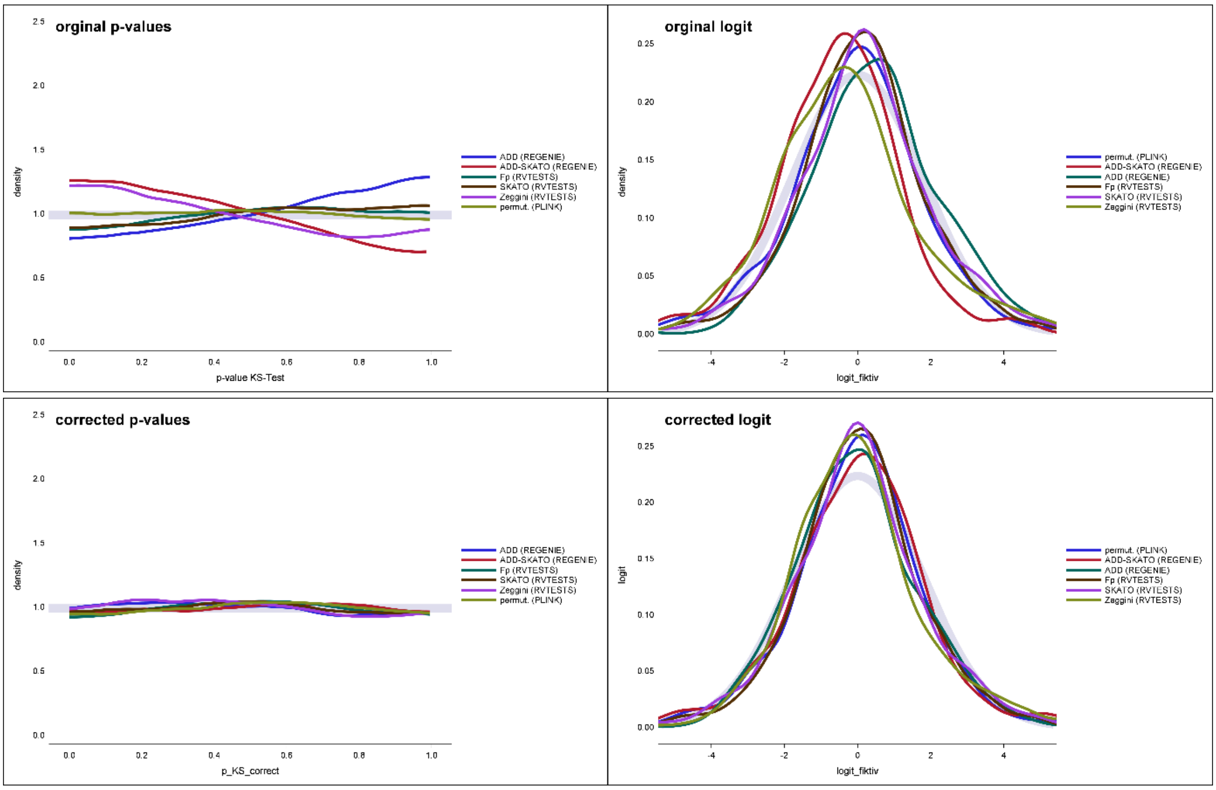

Leading-edge genes

S-Table 1 Leading-edge genes of at least suggestively significant HPO-terms

GENE	MINIMAL P-VALUE†	TEST	HPO NO.	-	-	-	-	-
ENO3	0.003	RVTESTS-Zeggini	HPO:0003326					
APC	0.005	RVTESTS-Fp	HPO:0003326	HPO:0004375	HPO:0040277	HPO:0100006		
CYP11B1	0.005	PLINK-permutation	HPO:0003154	HPO:0011043				
GMPPA	0.01	RVTESTS-Fp	HPO:0003154	HPO:0011043				
LPIN2	0.01	REGENIE-ADD	HPO:0003326					
ACAD9	0.02	RVTESTS-SKATO	HPO:0003326					
BRAF	0.02	RVTESTS-Zeggini	HPO:0003154	HPO:0011043	HPO:0040277			
FKTN	0.02	RVTESTS-Fp	HPO:0003326					
MAPT	0.02	RVTESTS-SKATO	HPO:0004375	HPO:0100006				
POR	0.02	RVTESTS-Fp	HPO:0003154	HPO:0011043				
RNASEH1	0.02	RVTESTS-SKATO	HPO:0003326					
SMO	0.02	RVTESTS-Fp	HPO:0002920	HPO:0004375	HPO:0011043	HPO:0040277	HPO:0100006	
ACP5	0.03	REGENIE-SKATO	HPO:0003326					
BDNF	0.03	RVTESTS-Zeggini	HPO:0004375	HPO:0100006				
DMD	0.03	PLINK-permutation	HPO:0003326					
PTPN2	0.03	RVTESTS-Fp	HPO:0003326					
ATP2A1	0.04	PLINK-permutation	HPO:0003326					
CAPN3	0.04	REGENIE-ADD	HPO:0003326					
DYSF	0.04	RVTESTS-SKATO	HPO:0003326					
ALX3	0.05	RVTESTS-SKATO	HPO:0004375	HPO:0100006				
CLCNKB	0.05	RVTESTS-Zeggini	HPO:0003326					
DSE	0.05	REGENIE-ADD	HPO:0003326					
PRKAR1A	0.05	PLINK-permutation	HPO:0002920	HPO:0004375	HPO:0011043	HPO:0040277		
WDPCP	0.05	PLINK-permutation	HPO:0004375	HPO:0100006				
ACADM	0.06	REGENIE-SKATO	HPO:0003326					
ANO5	0.06	RVTESTS-Zeggini	HPO:0003326					
CAVIN1	0.06	REGENIE-SKATO	HPO:0003326					
CLCN1	0.06	REGENIE-ADD	HPO:0003326					
LHX3	0.06	PLINK-permutation	HPO:0002920	HPO:0011043				
FGFR1	0.07	RVTESTS-Fp	HPO:0004375	HPO:0100006				
NBN	0.07	RVTESTS-Zeggini	HPO:0004375	HPO:0100006				
OTX2	0.07	REGENIE-ADD	HPO:0002920	HPO:0011043				
PDGFRA	0.08	REGENIE-ADD	HPO:0003326					
TSC2	0.08	RVTESTS-Zeggini	HPO:0004375	HPO:0040277	HPO:0100006			
AAGAB	0.09	PLINK-permutation	HPO:0004375	HPO:0100006				
BAP1	0.09	RVTESTS-Fp	HPO:0002920	HPO:0004375	HPO:0011043	HPO:0040277	HPO:0100006	
CRPPA	0.09	REGENIE-ADD	HPO:0003326					
KRAS	0.09	RVTESTS-SKATO	HPO:0004375	HPO:0040277	HPO:0100006			
NF1	0.09	REGENIE-SKATO	HPO:0004375	HPO:0100006				
PIK3CA	0.09	REGENIE-SKATO	HPO:0004375	HPO:0100006				
RB1	0.09	REGENIE-SKATO	HPO:0004375	HPO:0100006				
SCN4A	0.09	REGENIE-ADD	HPO:0003326					
STIM1	0.09	RVTESTS-SKATO	HPO:0003326					
ZSWIM6	0.09	REGENIE-SKATO	HPO:0004375	HPO:0100006				
CASQ1	0.10	REGENIE-SKATO	HPO:0003326					
CDKN1B	0.10	REGENIE-ADD	HPO:0040277					
SH3TC2	0.10	REGENIE-ADD	HPO:0003326					
COMT	0.11	REGENIE-SKATO	HPO:0003326					
EP300	0.11	RVTESTS-Fp	HPO:0004375	HPO:0100006				
GLI2	0.11	RVTESTS-Fp	HPO:0002920	HPO:0011043				
HRAS	0.11	PLINK-permutation	HPO:0004375					
LBR	0.11	RVTESTS-Zeggini	HPO:0003326					
NF2	0.11	RVTESTS-Fp	HPO:0002920	HPO:0004375	HPO:0011043	HPO:0040277	HPO:0100006	
SETBP1	0.11	RVTESTS-Fp	HPO:0004375	HPO:0100006				
TRAPPC11	0.11	REGENIE-SKATO	HPO:0003154	HPO:0003326	HPO:0011043			
AIP	0.12	REGENIE-ADD	HPO:0002920	HPO:0003154	HPO:0003326	HPO:0011043	HPO:0040277	
GNAS	0.12	RVTESTS-SKATO	HPO:0002920	HPO:0003154	HPO:0004375	HPO:0011043	HPO:0040277	HPO:0100006
PFKM	0.12	RVTESTS-Zeggini	HPO:0003326					
ALDH4A1	0.13	RVTESTS-Zeggini	HPO:0003326					
ASXL1	0.13	PLINK-permutation	HPO:0003326	HPO:0004375	HPO:0100006			
TBX1	0.13	REGENIE-SKATO	HPO:0003326					
PMP22	0.14	RVTESTS-SKATO	HPO:0003326					
ACADVL	0.15	RVTESTS-Fp	HPO:0003326					
ATRX	0.15	RVTESTS-Zeggini	HPO:0003154	HPO:0011043	HPO:0040277			
L2HGDH	0.15	RVTESTS-Fp	HPO:0004375					
SLC25A4	0.15	PLINK-permutation	HPO:0003326					
SMARCB1	0.15	REGENIE-ADD	HPO:0002920	HPO:0011043	HPO:0040277			
ALDOA	0.16	RVTESTS-Zeggini	HPO:0003326					
BIN1	0.16	REGENIE-SKATO	HPO:0003326					
DICER1	0.16	RVTESTS-Zeggini	HPO:0004375	HPO:0100006				
FAT4	0.16	RVTESTS-SKATO	HPO:0004375	HPO:0100006				
KIF1B	0.16	REGENIE-ADD	HPO:0004375	HPO:0100006				
PHKA1	0.16	PLINK-permutation	HPO:0003326					
AAAS	0.17	RVTESTS-Fp	HPO:0003154	HPO:0011043				
VCP	0.17	RVTESTS-Zeggini	HPO:0003326	HPO:0004375	HPO:0100006			

GENE	MINIMAL P-VALUE[†]	TEST	HPO NO.	-	-	-	-	-
**ZNF469**	0.17	REGENIE-ADD	HPO:0003326					
**ANKRD55**	0.18	RVTESTS-SKATO	HPO:0003326					
**DNA2**	0.18	REGENIE-ADD	HPO:0003326					
**NAB2**	0.18	RVTESTS-Fp	HPO:0004375					
**VHL**	0.18	PLINK-permutation	HPO:0004375	HPO:0100006				
**DNAJB6**	0.19	RVTESTS-SKATO	HPO:0003326					
**FLI1**	0.19	PLINK-permutation	HPO:0004375	HPO:0100006				
**LDHA**	0.19	PLINK-permutation	HPO:0003326					
**TP53**	0.19	REGENIE-ADD	HPO:0003154	HPO:0011043	HPO:0040277			
**GCDH**	0.21	RVTESTS-Zeggini	HPO:0004375	HPO:0100006				
**POLD1**	0.21	RVTESTS-Fp	HPO:0004375	HPO:0040277	HPO:0100006			
**SPTA1**	0.21	RVTESTS-Fp	HPO:0003326					
**SPRED1**	0.22	RVTESTS-Fp	HPO:0004375	HPO:0100006				
**TSC1**	0.25	PLINK-permutation	HPO:0004375	HPO:0100006				
**SEC23B**	0.26	RVTESTS-Fp	HPO:0004375	HPO:0100006				
**CDKN1A**	0.28	RVTESTS-Zeggini	HPO:0004375	HPO:0040277	HPO:0100006			
**POLE**	0.28	RVTESTS-Fp	HPO:0004375	HPO:0040277				
**PKD2**	0.29	RVTESTS-Fp	HPO:0040277					
**COQ6**	0.33	PLINK-permutation	HPO:0004375	HPO:0040277	HPO:0100006			
**ELP1**	0.35	RVTESTS-Zeggini	HPO:0004375					
**SDHA**	0.35	RVTESTS-SKATO	HPO:0003326	HPO:0004375	HPO:0100006			
**TK2**	0.35	RVTESTS-SKATO	HPO:0003326					
**ARMC5**	0.36	RVTESTS-Fp	HPO:0002920	HPO:0011043	HPO:0040277			
**DNM2**	0.36	REGENIE-SKATO	HPO:0003326					
**PNPLA2**	0.37	RVTESTS-SKATO	HPO:0003326					
**CCDC78**	0.39	RVTESTS-Fp	HPO:0003326					
**NOTCH3**	0.39	PLINK-permutation	HPO:0004375	HPO:0100006				
**ALG9**	0.40	PLINK-permutation	HPO:0040277					
**PHOX2B**	0.40	RVTESTS-Zeggini	HPO:0004375					
**EDN3**	0.45	RVTESTS-Fp	HPO:0004375	HPO:0100006				
**PTEN**	0.45	REGENIE-SKATO	HPO:0004375					
**MYO1H**	0.46	RVTESTS-Fp	HPO:0004375	HPO:0100006				
**GDNF**	0.47	PLINK-permutation	HPO:0004375	HPO:0100006				
**TGFBR2**	0.48	RVTESTS-Fp	HPO:0004375	HPO:0040277	HPO:0100006			
**BMPR1A**	0.53	PLINK-permutation	HPO:0004375	HPO:0040277	HPO:0100006			
**ASCL1**	0.62	PLINK-permutation	HPO:0004375					

[†] minimal p-value of all gene-level tests applied; HPO Human Phenotype Ontology
